# Sharp rise in high-virulence Bordetella pertussis with macrolides resistance in Northern China

**DOI:** 10.1080/22221751.2025.2475841

**Published:** 2025-03-05

**Authors:** Yahong Hu, Lin Zhou, Qianqian Du, Wei Shi, Qinghong Meng, Lin Yuan, Huili Hu, Lijuan Ma, Dongfang Li, Kaihu Yao

**Affiliations:** a Key Laboratory of Major Diseases in Children, Ministry of Education, National Clinical Research Center for Respiratory Diseases, National Key Discipline of Pediatrics, Laboratory of Infection and Microbiology, Beijing PaediatricPediatric Research Institute, Beijing Children’s Hospital, Capital Medical University, National Center for Children’s Health, Beijing, People’s Republic of China.; b Department of Clinical Laboratory, Capital Institute of, Beijing, People’s Republic of China.; c Department of Pediatrics, Beijing Shijingshan Hospital, Shijingshan Teaching Hospital, Capital Medical University, Beijing, People’s Republic of China.; d BGI, Shenzhen, People’s Republic of China.

**Keywords:** *Bordetella pertussis*, Erythromycin resistance, *Ptxp3*, MT28, China

## Abstract

**Objective:**

To elucidate the evolution of antigen genotype and antimicrobial resistance distribution of *Bordetella pertussis* (*B. pertussis*) from 2019 to 2023 in northern China.

**Methods:**

Polymerase chain reaction (PCR) amplification and sequencing were utilized to identify the seven antigen genotypes (*ptxA, ptxC, ptxP, prn, fim2, fim3, tcfA*). E-test and Kirby-Bauer (K-B) disc diffusion were employed to determine the minimum inhibitory concentration (MIC) and zone of inhibition for *B. pertussis* against antimicrobial agents. Subsequently, 50 isolates were chosen for multi-locus variable-number tandem-repeat analysis (MLVA) typing and whole-genome sequencing.

**Results:**

A total of 442 *B. pertussis* isolates were determined. The strains with high virulence harbouring *ptxP3* allele surged from 13.5% (21/155) in 2019–2021 to 93.0% (267/287) in 2022–2023. Concurrently, the erythromycin resistance *B. pertussis* (ERBP) in *ptxP3* isolates markedly rose from 42.9% (9/21) in 2019–2021 to 100% (267/267) in 2022–2023. The majority of *ptxP3* isolates (76.0%,219/288) exhibited the *ptxA1/ptxC1/prn2/fim2-1/fim3A/tcfA-2* genotype. Among the 442 confirmed patients, the children aged 3–14 years escalated rapidly from 13.5% in 2019 to 45.6% in 2023. The MT28 strains were responsible for 66.0% (33/50) of the tested ones, in which ERBP was prevalent at 87.9% (29/33). All the present sequenced *ptxP3*-ERBP strains (31/31) were clustered into the sub-lineage IVd.

**Conclusions:**

These results suggested the clonal spread of the *ptxP3*-ERBP lineage of *B. pertussis* with high virulence and macrolides resistance could be an important cause of the recent pertussis resurgence in China. Furthermore, the increased cases among pre-school and school-aged children underscore the importance of booster vaccination in this population.

## Introduction

Recently, there has been a sharp increase in pertussis cases worldwide, posing a significant public health concern. European countries such as the UK and Denmark have seen a surge in cases [1,2], with the Czech Republic experiencing its worst pertussis epidemic in a decade [3]. According to the monthly report released by the National Disease Control and Prevention Administration of China, the number of national reported pertussis cases has been steadily rising. Starting from 1512 cases in June 2023, the number surged to 9,126 cases in December, subsequently, there was a steep escalation to 15,275 and 17,105 cases in January–February 2024, followed by a dramatic surge to 27,078 cases in March and 91272 cases in April. It is concerning to note that pertussis is a highly contagious respiratory disease, leading to its continued spread and a much larger affected population. Pertussis is a vaccine-preventable disease, and since the 1950s, the diphtheria, tetanus, and pertussis vaccine (DTP) has been widely administered globally. The average global vaccination rate for three doses of pertussis vaccine reached 85.0% in 2019 [4]. However, over the past decade, there has been a resurgence of pertussis in some countries and regions with high vaccination rates [5,6].

The research on the pertussis resurgence found the distribution of antigen genotype of *Bordetella pertussis* continued to evolve. Presently, the most prevalent alleles are *ptxP1* and *ptxP3*, with *ptxP3* strains emerging as the dominant global strain due to their higher production of pertussis toxin (PT) compared to *ptxP1* strains [7]. The *ptxP3* strain is also frequently associated with increased variability in vaccine antigens, including pertactin-deficient variants [8], which may render it more susceptible to immune evasion by vaccines. Numerous prior studies in China, however, revealed that clinical isolates from 2000 to 2018 were predominantly comprised of strains harbouring the *ptxP1* allele, constituting between 78.9% and 94.9% [9–11]. Moreover, the majority of *ptxP1* strains were identified as the erythromycin resistance *B. pertussis* (ERBP). During the same period, *ptxP3* strains were more commonly isolated in certain local investigations in Shanghai and Shenzhen, accounting for 41.1% to 62.0% of cases, all of which were determined to be erythromycin sensitive [12,13]. Wu et al. reported two ERBP isolates expressing *ptxP3* in an investigation from 2017 to 2019, marking the first identification of such strains in China [14]. Subsequently, research conducted by Fu et al. revealed the emergence of an epidemic of *ptxP3* ERBP in Shanghai during 2021–2022 [15]. To comprehend the evolutionary patterns of antibiotic resistance and genotype distribution in the *B. pertussis* population on a larger scale, this study collected 442 culture-positive *B. pertussis* strains in northern China from 2019 to 2023, spanning three distinct phases before and after the COVID-19 pandemic outbreak. By integrating our findings with previous studies, we aim to conduct a comprehensive analysis of *B. pertussis* antimicrobial susceptibility and antigenic genotypes. This combined analysis will deepen our understanding of the epidemiological evolution of antimicrobial susceptibility and antigenic genotypes in *B. pertussis*, providing valuable scientific data and a solid foundation for controlling and containing the ongoing severe pertussis epidemic.

## Objects and methods

### Bacterial strains, patient demographics, and clinical information

From May 2019 to December 2023, nasopharyngeal swabs were collected from children suspected of pertussis visiting Beijing Children's Hospital and Children's Hospital, Capital Institute of Paediatrics. These samples were sent to the laboratory for *B. pertussis* isolation. Basic demographic information of the hosts, including age, gender, and residential location, was available for analysis. Additionally, clinical characteristics, including cough symptoms, duration, family history of cough exposure, and laboratory test results, were recorded for 100 hospitalized patients from Beijing Children's Hospital, for whom complete case data were available through the local electronic medical record system.

This study was approved by the Ethics Committee of Beijing Children's Hospital, Capital Medical University, with the ethics approval number [2022]-E-008-Y.

### Culture and identification

Nasopharyngeal swabs were cultured on plates with *B. pertussis* selective medium (OXOID, UK) supplemented with 10% defibrinated sheep blood and Bordetella selective supplement (OXOID, UK). Incubation was carried out at 35–37°C for 7 days, with daily monitoring of bacterial growth. Suspicious colonies appearing after 72 h were subjected to identification via slide agglutination test (Remel Europe Ltd., UK) using specific antisera for *B. pertussis* and *B. parapertussis*. In cases of indeterminate slide agglutination reactions, isolates underwent testing with a matrix-assisted laser desorption ionization-time-of-flight mass spectrometer (MALDI-TOF MS, Bruker, Germany). *B. pertussis* isolates were cryopreserved in bead storage tubes (PRO-LAB Microbank, Canada) at – 80°C.

### Antimicrobial susceptibility test

*B. pertussis* isolates were standardized to a McFarland standard of 0.5 and then inoculated onto charcoal agar supplemented with 10% sheep blood. Susceptibility to erythromycin, levofloxacin, ampicillin, and sulfamethoxazole/trimethoprim (SXT) was assessed using E-test strips (bio-Merieux, SA, France). Additionally, susceptibility to erythromycin and SXT was determined using Kirby-Bauer disc diffusion (Oxoid Ltd.,Basingstoke, United Kingdom), with verification by the E-test method. Minimum inhibitory concentration (MIC) and zone of inhibition diameter were measured after 96 h of bacterial culture. Quality control strains, *Staphylococcus aureus* ATCC 29213 and *Haemophilus influenzae* ATCC 49247, were utilized from the American Model Culture Collection Repository. As the American Clinical and Laboratory Standards Institute (CLSI) and the European Committee on Antimicrobial Susceptibility Testing (EUCAST) do not establish breakpoints for *B. pertussis*, susceptibility results are presented as MIC50, MIC90, MIC range, and zone of inhibition diameter range. Consistent with a previous study [14], erythromycin resistance breakpoints were defined as K-B disc inhibition diameter < 35 mm or MIC > 256 mg/L for this analysis.

### Sequence analysis of major antigen genes

Genomic DNA from *B. pertussis* isolates was extracted and purified using DNA extraction kits (Tiangen Biotechnology Co., Ltd., Beijing, China), following the manufacturer's instructions. Polymerase chain reaction (PCR) was employed to amplify seven virulence-related genotypes (*ptxA, ptxC, ptxP, prn, fim2, fim3, and tcfA*), which were subsequently sequenced as per established protocols [16]. The sequences were compared to known type sequences using BLAST software (http://www.ncbi.nlm.nih.gov/blast/Blast.cgi).

### Multiple loci variable-number tandem repeat (VNTR) analysis (MLVA), whole-genome sequencing, and phylogenetic analysis

We initially selected 10 strains of *B. pertussis* with different genotypes and erythromycin susceptibility to one another. This selection ensured diversity in genotypic profiles, with a particular emphasis on patterns of antibiotic resistance. Furthermore, an additional 40 strains were randomly selected based on temporal distribution and geographic origin of the patient to ensure broad representation in the study. This resulted in a total of 50 strains for MLVA and genome sequencing, with 39 strains carrying the *ptxP3* allele and 11 strains carrying the *ptxP1* allele. MLVA typing followed the procedure outlined by Schouls et al. [17], utilizing six variable-number tandem-repeat sequences (VNTR 1, VNTR 3a, VNTR 3b, VNTR 4, VNTR 5, and VNTR 6) from the *B. pertussis* MLVA database (https://www.mlva.net). In our study, paired-end reads from each strain were mapped to the Tohama I reference genome (GenBank accession NC_002929.2) using SOAP2 [18]. The base coverage of each position of the Tohama I genome was assessed using an in-house C/C++ program. Bases with a quality score of <20 were filtered out. To validate the resulting non-redundant candidate SNPs in Tohama I and the alleles of the other genomes, the numbers of the most abundant (n1) and the second most abundant (n2) nucleotides at each SNP in each strain (counted according to the number of reads in each strain supporting the presence of the nucleotide) were examined. High-quality SNPs satisfied the following criteria: (i) the most abundant base was different from that in the reference genome, (ii) n1 + n2 ≥ 10, and (iii) n1/n2 ≥ 5. SNPs called in repetitive regions of the reference genome, defined as exact repetitive sequences of 25 bp in length, identified using either BLAST, RepeatMasker, or Trf were excluded [19,20]. If at least 95% of the strains had a non-redundant SNP in a certain position, it was included in the SNP set. For the details regarding phylogenetic analyses and whole-genome sequencing, we adopted the methodology described by Wu et al. [14] and incorporated the two *ptxP3*-ERBP isolates from their study into our analysis.

### Statistical analyses

Data were analyzed using the χ2 test or Fisher’s exact test, as appropriate. All statistical analyses were performed using the SPSS (IBM, Chicago, IL, United States) software package version 26.0. Count data are presented as numbers or percentages, while non-normally distributed data are presented as median (interquartile range, IQR). A two-sided *P*-value < 0.05 was considered statistically significant.

## Results

### B. pertussis isolates

**Figure 1.**
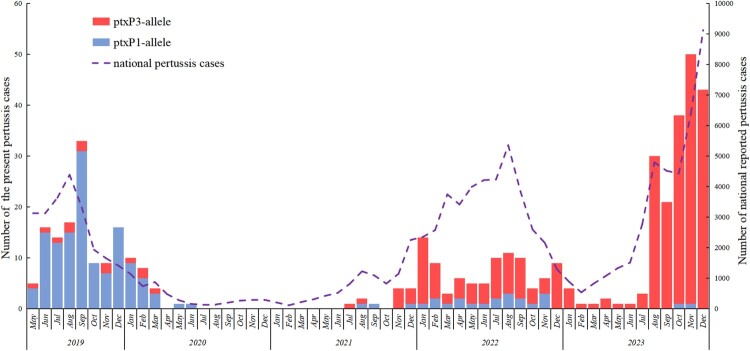
Monthly incidence of pertussis cases associated with *ptxP1* allele isolates and *ptxP3* allele isolates, and nationally reported pertussis cases spanning May 2019 through December 2023.

From May 2019 to December 2023, a total of 442 *B. pertussis* isolates were identified. The monthly distribution of these confirmed pertussis cases, along with the nationally reported number of pertussis cases (http://www.nhc.gov.cn/), is illustrated in Figure 1. Among the 442 cases, there were 119 in 2019 (26.9%), 24 in 2020 (5.4%), 12 in 2021 (2.7%), 92 in 2022 (20.8%), and 195 in 2023 (44.1%). Since the beginning of 2020, the detection of *B. pertussis* steadily declined to zero. From July 2020 to June 2021, no *B. pertussis* isolates were identified, resulting in a clear zero-detection phase in Figure 1. *B. pertussis* isolates were detected again starting from the second half of 2021, with a gradual increase in numbers thereafter. Throughout 2022, the number of isolates returned to levels consistent with regular patterns. However, in 2023, the number of pertussis cases began to surge, indicating an unusual trend of rapid increase. Overall, the monthly fluctuation in nationally reported pertussis cases in China during the study period closely mirrors that of the confirmed cases (Figure 1).

### Virulence-related genotypes

All present isolates were of *ptxA1/fim2-1/fim3A* alleles but showed variation in *ptxC, ptxP, prn, and tcfA* genotypes. Only two *ptxP* genotypes, *ptxP1* and *ptxP3*, were identified. The distribution of *ptxP1* and *ptxP3* in the isolates is illustrated in Figure 1. A comparison of the composition before and after the zero-detection phase reveals notable disparities. The ratio of *ptxP1* to *ptxP3* had completely reversed. The *ptxP1* isolates constituted 86.5% (134/155) of all isolates during 2019-2021, whereas the *ptxP3* isolates accounted for 93.0% (267/287) of all isolates during 2022–2023 (Table 1). None of the present isolates matched the genotype of the Chinese vaccine strain CS (*ptxA2/ptxC1/ptxP1/prn1/fim2-1/fim3A/tcfA-2*) in all alleles. In the present *ptxP1* isolates, genotype diversity was only found for *prn*, including 59.7% (92/154) *prn2* and 40.3% (62/154) *prn1*. The genotype diversity in *ptxP3* isolates, however, was found for *prn*, *ptxC,* and *tcfA*. Compared with the *ptxP1* isolates, the *ptxP3* ones are almost all *prn2* (99.7%, 287/288), and a significant portion of *ptxC2* (23.0%, 66/288).

**Table 1.** Genotypic characterization of 442 Bordetella pertussis Isolates in China from 2019 to 2023.

Genotype profile*	Total [*n* (%)]	Year distribution [*n* (%)]				
		2019	2020	2021	2022	2023
*ptxA1/ptxC1/ptxP1/prn1/fim2–1/fim3A/tcfA-2*	62(14.0)	25(21.0)	17(70.8)	3(25.0)	15(16.3)	2(1.0)
*ptxA1/ptxC1/ptxP1/prn2/fim2–1/fim3A/tcfA-2*	92(20.8)	85(71. 4)	4(16. 7)	0	3(3.3)	0
*ptxA1/ptxC1/ptxP3/prn2/fim2–1/fim3A/tcfA-2*	219(49.5)	9(7.6)	3(12.5)	9(75.0)	67(72.8)	131(67.2)
*ptxA1/ptxC1/ptxP3/prn4/fim2–1/fim3A/tcfA-2*	1(0.2)	0	0	0	1(1.1)	0
*ptxA1/ptxC1/ptxP3/prn2/fim2–1/fim3A/tcfA-5*	2(0.5)	0	0	0	0	2(1.0)
*ptxA1/ptxC2/ptxP3/prn2/fim2–1/fim3A/tcfA-2*	66(14.9)	0	0	0	6(6.5)	60(30.8)
Total	442	119	24	12	92	195

*Genotype of vaccine strain in China: *ptxA2/ptxC1/ptxP1/prn1/fim2-1/fim3A/tcfA-2*

### Antimicrobial susceptibility

The results of the antimicrobial susceptibility tests are presented in Table 2. Out of the 442 isolates tested, 427 (96.6%) exhibited a MIC of >256 mg/L and no inhibition zone against erythromycin, whereas the remaining 15 (3.4%) displayed MIC values ranging from 0.016–0.25 mg/L and inhibition zone diameters >55 mm. The rate of erythromycin resistance rose from 90.3% (140/155) in 2019–2021 to 100% (287/287) in 2022–2023. In particular, the *ptxP3* strains showed a concerning rise in erythromycin resistance, increasing from 42.9% (9/21) in 2019–2021 to 100% (267/267) in 2022–2023. Most isolates showed low MIC values for ampicillin (ranging from 0.094–32 mg/L, with an MIC90 of 0.5 mg/L) and levofloxacin (ranging from 0.094 to 1.5 mg/L). One isolate was determined high MIC value to ampicillin (>32 mg/L). Most isolates exhibited low MIC values or a wide diameter to SXT, while a small proportion had MIC values of ≥2 mg/L (1.1%, 5/442). Among these isolates, it was observed that the maximum MIC against SXT increased from 4 mg/L in 2019–2021 to 6 mg/L in 2022–2023.

**Table 2.** Antimicrobial susceptibility of the 442 Bordetella pertussis isolates in China during 2019–2023.

	Antibiotic	E-test (μg/ml)				Kirby–Bauer disk diffusion (mm)	
		MIC_50_^a^	MIC_90_^b^	MIC range	R (%)	Range of inhibition zone	R (%)
*ptxP1* (*n* = 154)	erythromycin	>256	>256	0.032 – >256	98.1	6–60	98.1
	Ampicillin	0.38	0.5	0.125-0.75	–	–*	–
	Levofloxacin	0.25	0.38	0.094-1.5	–	–	–
	Sulfamethoxazole/trimethoprim	0.25	0.75	0.006-4	4.5	10–65	10.4
*ptxP3* (*n* = 288)	erythromycin	>256	>256	0.016->256	95.8	6–64	95.8
	Ampicillin	0.25	0.5	0.094-32	–	–	–
	Levofloxacin	0.19	0.38	0.094-0.5	–	–	–
	Sulfamethoxazole/trimethoprim	0.047	0.38	0.006-6	1.7	10–65	1.4
Total (*n* = 442)	erythromycin	>256	>256	0.016->256	96.6	6–64	96.6
	Ampicillin	0.38	0.5	0.094-32	–	–	–
	Levofloxacin	0.25	0.38	0.094-1.5	–	–	–
	Sulfamethoxazole/trimethoprim	0.094	0.5	0.006-6	2.7	10–65	4.5

*: not tested in this study; R: resistant.

^a^ MIC50: 50th percentile of MIC values.

^b^ MIC90: 90th percentile of MIC values.

### MLVA and whole-genome sequencing

Among the 50 strains analysed, nine MLVA types were identified: MT16 (1 strain), MT22 (1 strain), MT27 (4 strains), MT28 (33 strains), MT55 (1 strain), MT76 (1 strain), MT104 (1 strain), MT195 (7 strains), and a novel type designated MT (1 strain). The novel MT shared 6 VNTRs (VNTR 1, VNTR 3a, VNTR 3b, VNTR 4, VNTR 5, and VNTR 6) with unit point variants of MT 195. All sequenced ERBP isolates (41/41) were confirmed to harbour the A2047G mutation within the 23S rRNA gene, whereas none of the erythromycin-susceptible isolates (0/9) exhibited this mutation.

Notably, MT28 was the predominant MLVA type, representing 66.0% (33/50) of the sequenced strains, among which MT28-ERBP was the most prevalent, accounting for 87.9% (29/33). Furthermore, the predominant antigenic genotype observed was *ptxP3/prn2* (76.0%, 38/50), encompassing both MT27 and MT28, with MT28 comprising the majority (89.5%, 34/38). All MT27 strains exhibited susceptibility to macrolides (100%, 4/4), whereas all MT195 strains carried the *ptxP1/prn1* genotype.

**Figure 2.**
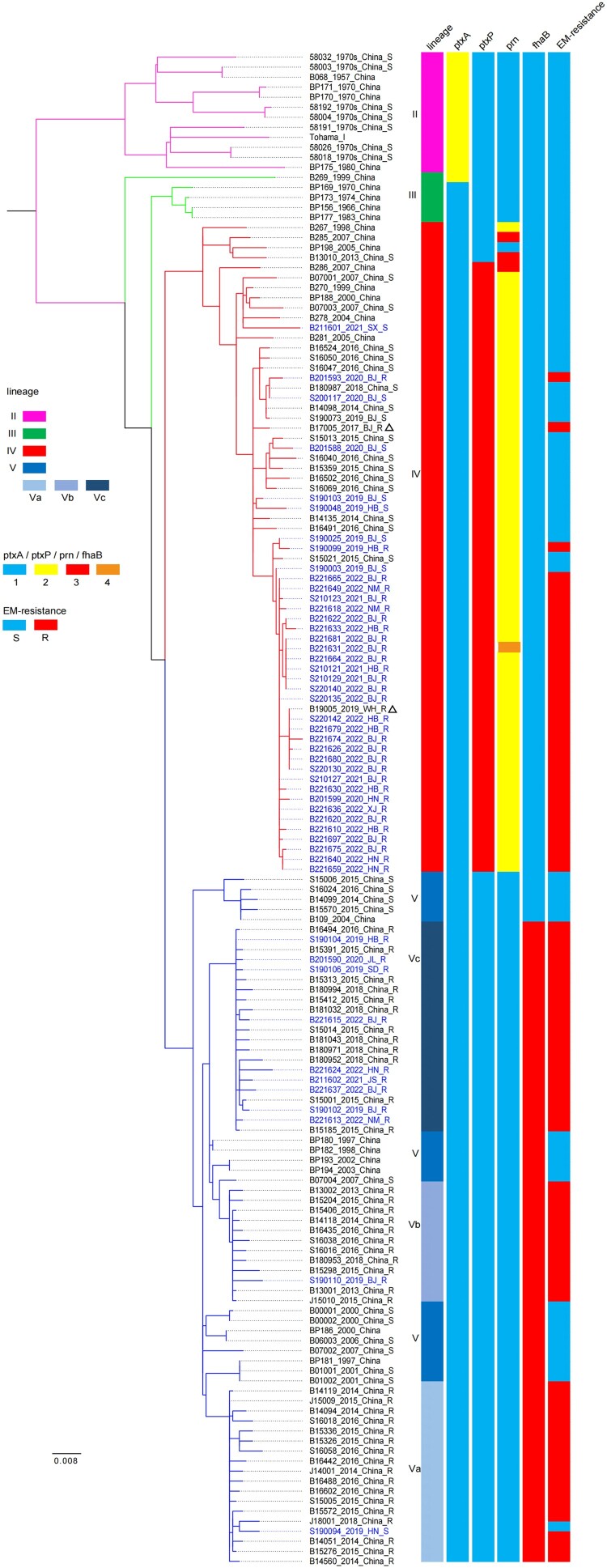
Construction of a maximum likelihood phylogenetic tree of Chinese *B. pertussis* isolates using genome-wide single nucleotide polymorphisms (SNPs). Leaves are labelled with “sample ID_isolate year_region_erythromycin resistance”. The leaf representing the studied strain is coloured blue. Two triangle-marked leaves represent the two *ptxP3*-ER (erythromycin-resistant) strains first discovered in China. Vertical bars on the right indicate the pedigree, *ptxA, ptxP, prn, fhaB* allele types, and erythromycin resistance (ER) for each strain or subline. Legend located on the left side of the figure.

Based on the determined 1085 SNPs, a phylogenetic tree was constructed and shown in Figure 2. As expected, the 50 isolates sequenced in this present study are classified into lineage IV or V. The eleven isolates carrying the *ptxP1* gene were categorized into lineage V, predominantly sub-lineage Vc, while the remaining 39 strains, harbouring the *ptxP3* gene, were assigned into lineage IV. Genome analyses revealed that all *ptxP3-*ERBP carried the *fhaB-1* allele, whereas *ptxP1-*ERBP isolates carried the *fhaB-3* allele. Supplementary figure 1 was constructed based on the 1503 SNPs in lineage IV, which demonstrated that strains within lineage IV share close genetic relationships, irrespective of their geographic origin, and the present *ptxP3*-ERBP isolates in lineage IV, including the present 31 *ptxP3*-ERBP ones were all assigned into sub-lineage IVd. Furthermore, analysis of the sequenced genomes from the 50 *B. pertussis* isolates did not confirm the presence of a reversed *IS481* insertion in the *prn* locus, nor did it reveal any evidence of *fhaB* deficiency. The whole-genome sequences and complete genome sequences were deposited in the NCBI Sequence Read Archive, BioProject: PRJNA1155883.

### Clinical data

**Figure 3.**
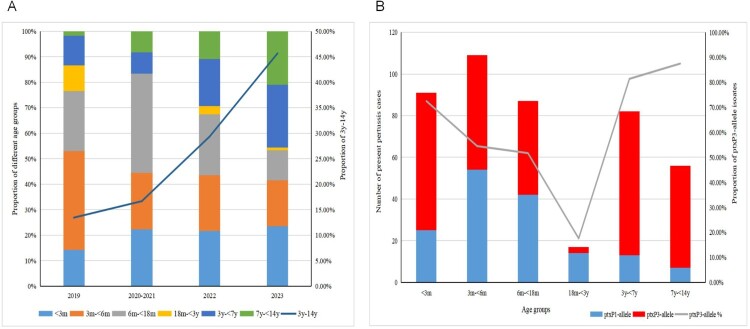
(A) Age distribution of the present pertussis cases and the trend of the proportion of 3y-14y from 2019 to 2023. (B) The numbers of the present pertussis cases and the proportion of *ptxP3* allele isolates by age groups.

The age distribution of the 442 patients revealed that 91 (20.6%) were < 3 months old, 109 (24.7%) were between 3 and <6 months, 87 (19.7%) were between 6 months and <18 months, 17 (3.9%) were between 18 months and <3 years, 82 (18.6%) were between 3 years and <7 years, and 56 (12.7%) were between 7 years and <14 years (Supplementary Figure 2). The annual proportion of children aged 3 to <14 years exhibited an increasing trend: 13.5% in 2019, 16.67% in 2020-2021, 29.4% in 2022, and 45.6% in 2023 (Figure 3A). The *ptxP3* isolates were more frequently observed in the 3 to <7 years age group (81.4%) and the 7 to <14 years age group (87.5%) compared to other younger age groups (17.6%−72.5%) (Figure 3B). Of the 442 patients, 245 were male and 197 were female.

Among the 100 patients with accessible medical records, before sampling for *B. pertussis* culture, 52.0% (52/100) of patients had a cough lasting more than two weeks, 35.0% (35/100) lasted >1-<2 weeks, and the remainder (13.0%, 13/100) < 1 week. Additionally, Twenty-eight cases (28.0%, 28/100) had a history of exposure to individuals with a cough, primarily parents or siblings. Eighteen patients had a fever; however, 50.0% (9/18) of them experienced temporary fever peaking at (38.5 ± 0.7) °C. Recurrent fever was recorded in three cases, while six cases exhibited persistent fever. Only one death case was confirmed, which was a 42-day-old girl, caused by a *ptxP3* isolate. Cases of pertussis caused by *ptxP3* strains were more likely to exhibit paroxysmal cough (*P *= 0.026) and fever (*P *= 0.039) compared to those infected with *ptxP1* strains (Supplementary tables 1). Meanwhile, infants under 6 months old infected with pertussis caused by *ptxP3* strains were significantly more likely to experience respiratory failure (*P *= 0.034) compared to those infected with *ptxP1* strains (Supplementary tables 2 and 3).

## Discussion

In the past decade, ERBP has been frequently identified in China, initially without exception identified as *ptxP1* isolates until 2017 [21,22]. Despite *ptxP3* isolates being determined at various frequencies (ranging from 10.7% to 46.7%) [10,23], they remained sensitive to erythromycin. Wu et al. identified the first two *ptxP3*-ERBP in China: one isolated from a child in Jiangsu province in 2017 and the other from Anhui province in 2019 [14]. These strains belonged to lineage IV and had different MLVA types-MT27 and MT28, respectively. Since similar *ptxP3* strains have already spread internationally despite varying immunization selection pressures, the authors speculated that *ptxP3*-ERBP would soon spread in China, posing a potentially significant challenge for pertussis prevention and control worldwide [14].

The present results and several recent studies suggest that this earlier assumption is reasonable. The present MT-28 *ptxP3*-ERBP strains are very close to one of them (B19005_2019_WH_R) from Anhui in the phylogenetic tree (Figure 2). The *ptxP3* strains subversively replaced *ptxP1* strains by 93.0% (267/287) during 2022-2023, with 100% (267/267) of ERBP. The MT28 was the predominant type (66.0%, 33/50) in the tested strains, and the majority of MT28 strains belonging to the *ptxP3*-ERBP (58.0%, 29/50). Several recent studies in China revealed similar findings. Zhou et al. demonstrated that *ptxP3* strains show a high level of macrolide resistance, which has become a major challenge for clinical treatment [24]. Similarly, Fu et al. reported that *ptxP3*-ERBP strains comprised 62% of clinical Bordetella pertussis isolates in Shanghai between 2021 and 2022 [15]. They further indicated that the post-COVID-19 resurgence of pertussis was closely linked to the spread of the *ptxP3* macrolide-resistant MT28 clone [25]. Moreover, Cai et al. identified a novel erythromycin-resistant MT28 clone from sub-lineage IVd, which has accelerated the transition from *ptxP1* to *ptxP3* in Shanghai [26].

The present results underscore the distinct epidemiological characteristics of pertussis in China compared to most developed countries. In these countries [27,28], the predominant strain is usually MT27 carrying the *ptxP3* allele, and MT28 strains typically remain susceptible to erythromycin. Previous studies conducted in Australia and Japan [29,30], have demonstrated that *ptxP3* strains exhibit higher levels of PT expression compared to *ptxP1* strains. Furthermore, these studies suggest that *ptxP3* strains may possess a competitive edge over *ptxP1* strains when subjected to vaccine-induced selection pressure. In line with these findings, Safarchi et al. demonstrated that *ptxP3* strains exhibit increased fitness in mice vaccinated with the acellular vaccine compared to *ptxP1* strains [31]. Moreover, the prevalence of *ptxP3/prn2*, MT28, and Spectrum IV types further illustrates the robust adaptability of *ptxP3* strains to immune selection pressure [14]. In China, a substantial epidemiological transition has been documented in *B. pertussis* strain dynamics during the past two decades. Molecular epidemiological surveillance data reveal that *ptxP3* strains maintained a low-prevalence endemic status throughout their pre-resistance phase. However, subsequent to the acquisition of macrolide resistance determinants, these strains exhibited enhanced biological fitness and transmission capacity, resulting in their rapid clonal expansion and subsequent displacement of *ptxP1*-ERBP strains within a remarkably compressed epidemiological timeframe. This emerging pattern of antimicrobial resistance and strain replacement poses substantial challenges for current pertussis prevention strategies, with significant implications for both national and global pertussis control programmes. During this strain replacement process, genomic analysis revealed that the emerging *ptxP3* strains maintain the *fhaB-1* allele, which is identical to that of the vaccine strain, whereas the displaced *ptxP1* strains predominantly carry the *fhaB-3* allele. This allelic distribution pattern suggests that the immunological selection pressure exerted by *fhaB-*specific immunity may play a less substantial role in strain evolution than we previously hypothesized [14].

Previous studies conducted overseas have indicated that strains carrying the *ptxP3* allele exacerbate pertussis symptoms and are linked to higher rates of hospitalization and mortality [29,30]. Conversely, prior studies conducted in China have demonstrated contradictory findings: cases attributed to *ptxP3* strains were less symptomatic than those attributed to *ptxP1* strains [14]. In the current study, cases attributed to *ptxP3* strains exhibited a higher susceptibility to complications such as pneumonia, respiratory failure, and myocardial injury, and demonstrated a higher incidence of respiratory failure compared to those attributed to *ptxP1* strains. This suggests that pertussis cases caused by *ptxP3* strains may be more severe. The present single fatal case was an infant aged 42 days caused by *ptxP3*-ERBP strain. This finding further corroborates our initial hypothesis that diseases caused by *ptxP3* strains (without erythromycin resistance) were milder due to the overuse of macrolides in China [14]. However, upon acquiring erythromycin resistance, the *ptxP3* isolates demonstrated greater pathogenicity than the *ptxP1* isolates. The clinical symptoms and disease severity observed in this study may have been influenced by additional factors, such as co-infections with other pathogens, which require further investigation.

The spread of *ptxP3*-ERBP strains with high virulence and drug resistance also be associated with increased pertussis cases in older children as shown in the present results. Investigations into the resurgence of pertussis in regions with high vaccination coverage, such as North America, Europe, and Asia, revealed a higher incidence of the disease in older children, adolescents, and adults [32,33]. However, infants have consistently been the primary age group affected since the reporting of pertussis cases began in China in 2013 [34,35]. This trend may now be shifting. The results of this study reveal a significant increase in the proportion of confirmed pertussis cases in children aged over 3 years, rising gradually from 13.5% (16/119) in 2019 to 45.6% (89/195) in 2023. This trend warrants attention. These percentages represent a significant increase compared to our previous report of 3.6% (12/335) between 2014 and 2016 [10]. To date, pertussis vaccination is not included in the national immunization programme in China after two years of age. Consequently, it was expected that more pertussis cases would be observed and reported in older children, as well as in adolescents and adults in the future. The increasing incidence of pertussis in elderly groups raises concerns about pertussis immunization in these populations.

During the COVID-19 epidemic, strict control measures including masking, containment management, entry and exit controls, and quarantine were rigorously implemented, particularly from 2020 to 2021. Consequently, there was a significant decline in the incidence of respiratory infectious diseases [36]. As a result, the number of reported pertussis cases per year in China plummeted from 30,027 in 2019 to 4,475 in 2020. However, this figure rebounded to 9,611 in 2021 and surged to 39,781 and 38,205 in 2022 and 2023, respectively (Figure 1). Explaining the epidemiological changes of pertussis during the COVID-19 control phase is challenging. This abnormal change may be due to the significant differences between pertussis and COVID-19, such as their disease progression, incubation periods, and population susceptibility. The short-term control measures for COVID-19, including home isolation, may have inadvertently facilitated the spread of pertussis during the later stages of the pandemic, as the isolation period may not have been long enough for patients to fully recover at home, potentially leading to increased internal pertussis transmission within households. The infected family members would initiate the community transmission when the controls ended.

SXT could serve as an alternative antibiotic for patients who have failed macrolide treatment [37]. Wu et al. and Fu et al. determined the minimum inhibitory concentration (MIC) of *B. pertussis* against SXT, ranging from 0.008 to 0.5 and 0.008 to 0.125, respectively [13,14], demonstrating notably low levels. However, the present investigation has revealed several isolates with MICs as high as 6 mg/L against SXT, suggesting that SXT antibiotic treatment may not effectively clear such isolates. The limited genomic data in this study cannot determine the genetic mechanism of the increase of MICs to SXT. This phenomenon warrants continuous surveillance, which will collect more samples with such character for further study.

Current clinical isolates of *B. pertussis* primarily originate from patients in northern China and may not adequately represent the national prevalence of the disease. However, residence information was available for the present 403 confirmed cases, with the majority residing in Beijing (*n* = 190), followed by Hebei (*n* = 151), Henan (*n* = 18), Inner Mongolia Autonomous Region (*n* = 12), Shandong (*n* = 6), Heilongjiang (*n* = 5), and other twelve administrative provinces (*n* ≤ 3). This study spanned five years and included a substantial number of strains, providing an accurate reflection of the evolutionary characteristics of *B. pertussis* in northern China.

In summary, the present results indicate a complete reversal in the compositional ratios of *ptxP1* and *ptxP3* in *B. pertussis* isolates, which coincided with the later stages of the COVID-19 pandemic and might be linked to the dissemination of *ptxP3*-ERBP in sub-lineage IVd. The clonal spread of *ptxP3*-ERBP lineage of *B. pertussis* with high virulence and macrolide resistance could be an important cause of the recent pertussis resurgence in China. The present data showed that infants continue to be the primary victims of pertussis in China. However, it is noteworthy that the older age groups are gradually replacing infants in terms of proportion. The increased cases among pre-school and school-aged children underscore the importance of booster vaccination in this population. There is a foreseeable increase in pertussis cases among older children, adolescents, and adults, highlighting the need for immunization in these age groups.

## Supplementary Material

Supplementary Figure1.jpg

supplementary tables.doc

Supplementary Figure2.jpg
