# Supplementary figures and images for: Sharp rise in high-virulence Bordetella pertussis with macrolides resistance in Northern China
Source: Emerg Microbes Infect. 2025 Mar 5;14(1):2475841. doi: 10.1080/22221751.2025.2475841 (PMC11921162; doi:10.1080/22221751.2025.2475841)

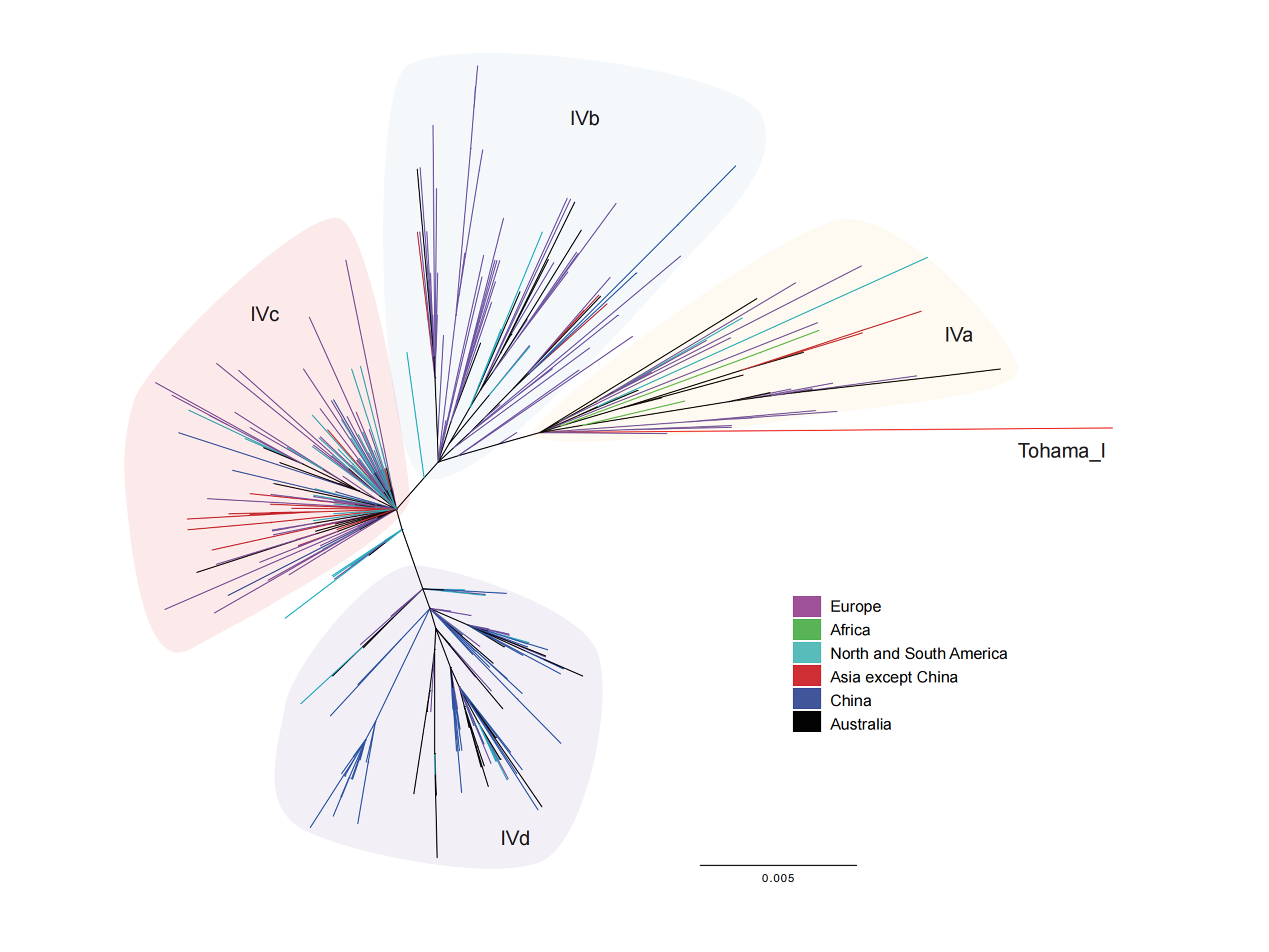

Supplement: Supplementary Figure1.jpg [file TEMI_A_2475841_SM8644.jpg]

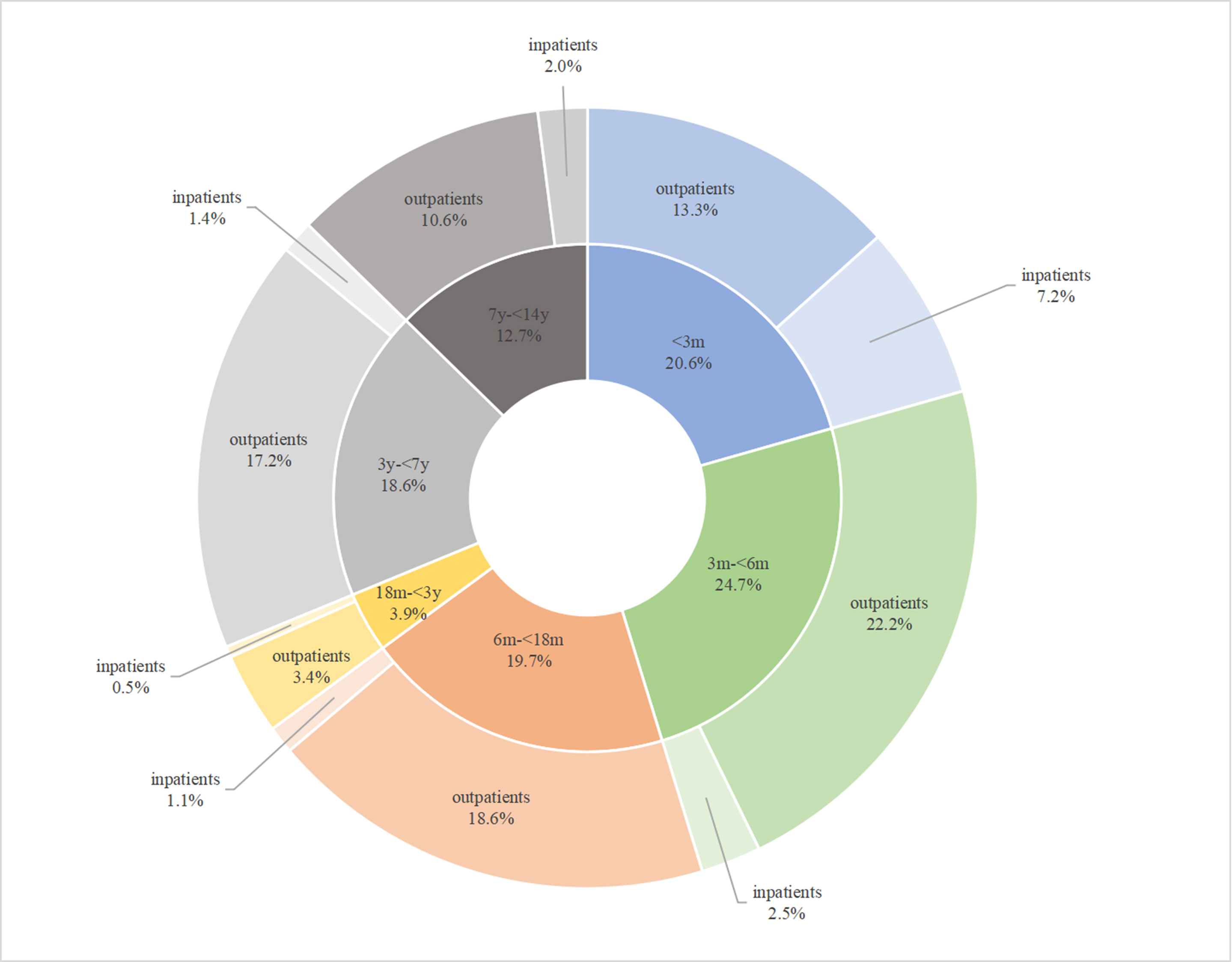

Supplement: Supplementary Figure2.jpg [file TEMI_A_2475841_SM8642.jpg]
