# Supplementary material for: Sharp rise in high-virulence Bordetella pertussis with macrolides resistance in Northern China
Source: Emerg Microbes Infect. 2025 Mar 5;14(1):2475841. doi: 10.1080/22221751.2025.2475841 (PMC11921162; doi:10.1080/22221751.2025.2475841)
Supplement: supplementary tables.doc [file TEMI_A_2475841_SM8643.doc]

| **Supplementary table 1. Comparison of clinical characteristics between the pertussis cases caused by *ptxP1* and *ptxP3* stains in China from 2019 to 2023** | | | | |  |
| --- | --- | --- | --- | --- | --- |
| **No. (%) patients** | | | | |  |
| Patient characteristic | Total patients  n = 100 | *ptxP3* Strains  n =62 | *ptxP1* Strains  n = 38 | P Value | |
| Cough |  |  |  |  | |
| Paroxysmal | 43(43.0) | 32(51.6) | 11(28.9) | 0.026 | |
| Spasmodic | 21(21.0) | 16(25.8) | 5(13.2) | 0.132 | |
| Post-tussive vomiting | 12(12.0) | 7(11.3) | 5(13.2) | 0.762 | |
| Excessive phlegm | 45(45.0) | 32(51.6) | 13(34.2) | 0.09 | |
| Fever | 18(18.0) | 15(24.2) | 3(7.9) | 0.039 | |
| Facial blushing | 24(24.0) | 14(22.6) | 10(26.3) | 0.671 | |
| Disease duration at admission(d) (IQR) | 14（8.3-18.8） | 14（8.75-15.0） | 11.5(7.8-28.5) | 0.937 | |
| Rhinorrhea | 15(15.0) | 11(17.7) | 4(10.5) | 0.327 | |
| Wheezing | 24(24.0) | 13(21) | 11(28.9) | 0.364 | |
| Apnea | 14(14.0) | 9(14.5) | 5(13.2) | 0.849 | |
| Respiratory Failure | 11(11.0) | 10(16.1) | 1(2.6) | 0.078 | |
| Complicated pneumonia | 79(79.0) | 59(96.2) | 20(52.6) | 0.606 | |
| Complication of severe pneumonia | 9(9.0) | 7(11.3) | 2(5.3) | 0.508 | |
| Extrapulmonary organs damage^a^ | 16(16.0) | 12(19.4) | 4(10.5) | 0.242 | |
| Death | 1(1.0) | 1(1.6) | 0(0) | NA* | |
| Younger than 3 months old, n (%) | 22(22.0) | 14(22.6) | 8(21.1) | 0.858 | |
| Older than 18 months old, n (%) | 29(29.0) | 23(37.1) | 6(15.8) | 0.023 | |
| Household contacts | 28(28.0) | 18(29) | 10(26.3) | 0.769 | |
| Laboratory findings |  |  |  |  | |
| Peak WBC count (×10^9^/L) (IQR) | 16.91 (11.2,25.4) | 17.94(11.5,26.5) | 15.43(10.5,23.8) | 0.534 | |
| LYMPH%(IQR) | 66.7(55.0,72.9) | 66.35(53.0,72.8) | 68.4(56.5,73.3) | 0.417 | |
| NEUT%(IQR) | 24.75(19.0,34.3) | 26.3(20.1,38.0) | 24.2(17.7,33.1) | 0.376 | |
| *NA, not analyzed.  ^a^Extrapulmonary organs involved in liver, heart, or brain here. | | | | | |

| **Supplementary table 2. Clinical characteristics of 43 pertussis patients under 6 months old, May 2019–December 2023** | | | | |  |
| --- | --- | --- | --- | --- | --- |
| **No. (%) patients** | | | | |  |
| Patient characteristic | Total patients  n = 43 | *ptxP3* Strains  n =24 | *ptxP1* Strains  n =19 | P Value | |
| Cough |  |  |  |  | |
| Paroxysmal | 16(37.2) | 10(41.7) | 6(31.6) | 0.497 | |
| Spasmodic | 9(20.9) | 7(29.2) | 2(10.5) | 0.265 | |
| Post-tussive vomiting | 7(16.3) | 4(16.7) | 3(15.8) | 1.0 | |
| Excessive phlegm | 17(39.5) | 10(41.7) | 7(36.8) | 0.748 | |
| Fever | 6(14.0) | 4(16.7) | 2(10.5) | 0.893 | |
| Facial blushing | 14(32.6) | 9(37.5) | 5(26.3) | 0.437 | |
| Disease duration at admission(d)(IQR) | 10（7.0-15.0） | 13(7.3-15.0) | 10（7.0-13.0） | 0.133 | |
| Rhinorrhea | 2(4.7) | 2(8.3) | 0 | NA* | |
| Wheezing | 9(20.9) | 7(29.2) | 7(36.8) | 0.594 | |
| Apnea | 9(20.9) | 6(25.0) | 3(15.8) | 0.719 | |
| Respiratory Failure | 10(23.3) | 9(37.5) | 1(5.3) | 0.034 | |
| Complicated pneumonia | 35(81.4) | 20(83.3) | 15(78.9) | 0.893 | |
| Complication of severe pneumonia | 6(14.0) | 4(16.7) | 2(10.5) | 1.0 | |
| Extrapulmonary organs damage^a^ | 9(20.9) | 6(25.0) | 3(15.8) | 0.719 | |
| Household contacts | 19(44.2) | 11(45.8) | 8(42.1) | 0.807 | |
| Laboratory findings |  |  |  |  | |
| Peak WBC count (×10^9^/L) (IQR) | 14.9 (11.2,22.5) | 14.9(10.2,23.3) | 14.9(11.3,20.2) | 0.694 | |
| LYMPH%(IQR) | 70.7(59.3,73.7) | 67.6(65.2,74.3) | 70.7(55.0,73.6) | 0.066 | |
| NEUT%(IQR) | 21.4(15.5,27.4) | 20.6(14.9,27.2) | 21.7(16.3,33.4) | 0.645 | |
| *NA, not analyzed.  ^a^Extrapulmonary organs involved in liver, heart, or brain here. | | | | | |

| **Supplementary table 3. Clinical characteristics of 57 pertussis patients older than 6 months old, May 2019–December 2023** | | | | |
| --- | --- | --- | --- | --- |
| **No. (%) patients** | | | | |
| Patient characteristic | Total patients  n = 57 | *ptxP3* Strains  n =38 | *ptxP1* Strains  n =19 | P Value |
| Cough |  |  |  |  |
| Paroxysmal | 27(47.4) | 22(57.9) | 5(26.3) | 0.024 |
| Spasmodic | 12(21.1) | 9(23.7) | 3(15.8) | 0.730 |
| Post-tussive vomiting | 5(8.8) | 3(7.9) | 2(10.5) | 1.0 |
| Excessive phlegm | 28(49.1) | 22(57.9) | 6(31.6) | 0.061 |
| Fever | 12(21.1) | 11(28.9) | 1(5.3) | 0.085 |
| Facial blushing | 10(17.5) | 5(13.2) | 5(26.3) | 0.389 |
| Disease duration at admission(d))(IQR) | 14.0（10.0,27.5） | 14.0(10.0,24.3) | 15.0(10.0,30.0) | 0.958 |
| Rhinorrhea | 13(22.8) | 9(23.7) | 4(21.1) | 1.0 |
| Wheezing | 10(17.5) | 6(15.8) | 4(21.1) | 0.902 |
| Apnea | 5(8.8) | 3(7.9) | 2(10.5) | 1.0 |
| Respiratory Failure | 2(3.5) | 2(5.3) | 0 | NA* |
| Complicated pneumonia | 44(77.2) | 30(78.9) | 14(73.7) | 0.911 |
| Complication of severe pneumonia | 3(5.3) | 3(7.9) | 0 | NA* |
| Extrapulmonary organs damage^a^ | 7(12.3) | 6(15.8) | 1(5.3) | 0.476 |
| Household contacts | 9(15.8) | 7(18.4) | 2(10.5) | 0.700 |
| Laboratory findings |  |  |  |  |
| Peak WBC count (×10^9^/L) (IQR) | 18.4(10.8,28.0) | 18.7(12.4,27.6) | 16.2(9.4,36.1) | 0.162 |
| LYMPH%(IQR) | 61.3(52.6,71.1) | 58.6(48.1,71.2) | 64.8(57.5,71.6) | 0.565 |
| NEUT%(IQR) | 29.9(22.6,38.3) | 33.7(22.8,40.3) | 28.6(18.8,32.6) | 0.689 |
| *NA, not analyzed.  ^a^Extrapulmonary organs involved in liver, heart, or brain here. | | | | |
